# Crystallization of *n*-Alkanes under Anisotropic Nanoconfinement in Lipid Bilayers

**DOI:** 10.1021/acs.jpcb.4c04332

**Published:** 2024-12-19

**Authors:** Anika Wurl, Maria Ott, Christian Schwieger, Tiago M. Ferreira

**Affiliations:** † NMR Group—Institute for Physics, Martin Luther University Halle-Wittenberg, Halle 06120, Germany; ‡ Department of Biotechnology and Biochemistry, Martin Luther University Halle-Wittenberg, Halle 06120, Germany; § Institute of Chemistry, Martin Luther University Halle-Wittenberg, Halle 06120, Germany; ∥ CiQUS and Department of Physical Chemistry, University of Santiago de Compostela, Santiago de Compostela 15705, Spain

## Abstract

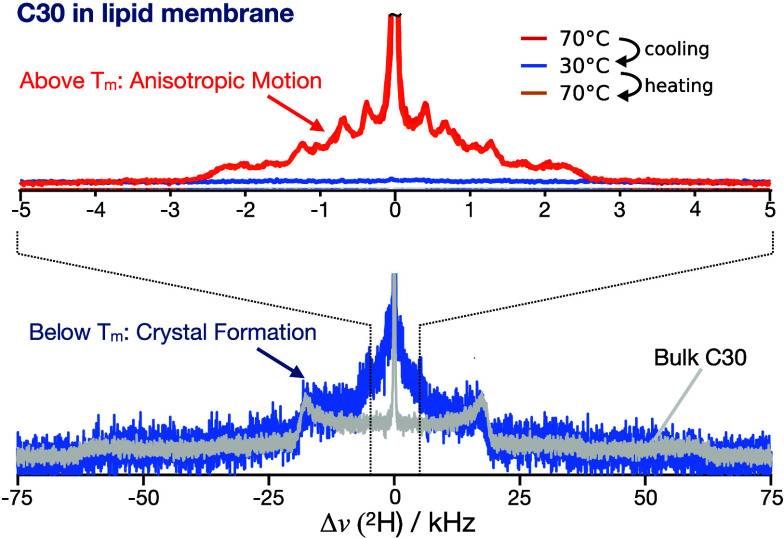

Understanding crystallization behavior is integral to the design of pharmaceutical compounds for which the pharmacological properties depend on the crystal forms achieved. Very often, these crystals are based on hydrophobic molecules. One method for delivering crystal-forming hydrophobic drugs is by means of lipid nanoparticle carriers. However, so far, a characterization of the potential crystallization of fully hydrophobic molecules in a lipid environment has never been reported. In this work we investigate the crystallization behavior of two model hydrophobic chains, *n*-eicosane (C20) and *n*-triacontane (C30), in phospholipid bilayers. We combine static ^2^H nuclear magnetic resonance (NMR) spectroscopy and differential scanning calorimetry (DSC) and show that C30 molecules can indeed crystallize inside DMPC and POPC bilayers. The phase transition temperatures of C30 are slightly reduced inside DMPC, and rotator phase formation becomes a two-step process: Preorganized *n*-alkane chains assemble in rotator-phase crystallites just as fast as bulk C30, but further addition of molecules is notably slower. Under the same isothermal conditions, different crystal forms can be obtained by crystallization in the membrane and in bulk. In excess water conditions, homogeneous nucleation of C30 is observed. The initial anisotropic molecular arrangement of C30 molecules in the membrane is readily recovered upon reheating, showing reversibility. The shorter C20 molecules on the other hand become trapped in the DMPC membrane gel-phase upon cooling and do not crystallize. This work marks the first observation of the crystallization of hydrophobic chains inside a lipid bilayer environment. As such, it defines a fundamental starting point for studying the crystallization characteristics of various hydrophobic molecules in lipid membranes.

## Introduction

From a biophysical perspective, the interactions between hydrophobic molecules and cell membranes are integral to a variety of processes, such as lipid droplet formation,^1−3^ the effect of micro- and nanoplastic pollution on living cells^4,5^ or nanoparticle design for drug delivery.^6−9^ Concerning the latter, various researchers have been exploring the possibility of using liposomes as drug delivery vehicles for hydrophobic drug molecules. Many of these drugs crystallize in aqueous media and have been typically administrated as stabilized nanocrystalline drug suspensions. The nanocrystals often show polymorphism, with the crystal properties strongly affecting drug stability and performance.^10^ Therefore, it is essential to determine if such drugs can also crystallize inside the lipid membrane vehicle, and if such a potential crystallization may be controlled by the composition of the nanocarriers. However, there is still a lack of research on allegedly simple scenarios. For example, to our knowledge, the potential crystallization of purely hydrophobic chain-like molecules inside a lipid membrane has never been reported.

The nanoconfinement realized by lipid bilayers presents a unique system that is of fundamental interest for crystallization studies, since the lipid membrane resembles an anisotropic solution featuring a gradient of molecular order. This type of confinement is substantially different from the confinement in emulsion droplets, nanoparticles or porous materials. The interaction of purely hydrophobic molecules with lipid membranes has been addressed in a number of simulation studies, many of which were inspired by the increasing accumulation of nanoplastics in the environment.^11−16^ However, there is a lack of experimental studies related to this topic, partly due to the fact that such systems are challenging to prepare experimentally. In contrast to the partial insertion of amphiphilic or polyphilic molecules, the addition of purely hydrophobic molecules often leads to pore formation in the membrane, or phase separation of the components.^17^ The first experimental studies date back to the 1980s with the work of Pope and co-workers on the inclusion of *n*-alkanes in lipid membranes.^18,19^ These studies suggested that *n*-alkanes with chain lengths higher than 18 carbons are nearly immiscible with the lipid acyl tails. Later, squalane has been found to incorporate in the bilayer center of model membranes^20^ and Bochicchio et al. demonstrated the effect of polystyrene 25-mers on dipalmitoylphosphatidylcholine (DPPC) membrane thermal and mechanical properties using calorimetry, X-ray and neutron scattering.^5^ Very recently, we showed with ^2^H nuclear magnetic resonance (NMR) that about 3–5 vol % of *n*-triacontane (C30) can be incorporated into DPPCor dimyristoylphosphatidylcholine (DMPC) membranes.^21^ This system is a perfect starting point for investigating the crystallization of hydrophobic chain-like molecules inside lipid membranes.

The crystallization behavior of bulk *n*-alkanes often comprises the existence of the so-called rotator phases, which may be formed prior to the crystalline phase upon cooling.^22,23^ These rotator phases are defined by a rotational freedom along the long axis of the molecules, while the positional long-range order is retained along all dimensions, analogously to what occurs in lipid gel phases that are formed between the liquid-crystalline and crystalline phases in lipid bilayers.^24^ The occurrence, stability and nature of the rotator phases strongly depends on the length of the *n*-alkanes, as has been reviewed in detail by a number of authors.^22,23,25^*n*-Alkanes with stable rotator phases crystallize via rotator phase nuclei, before transforming into the low-temperature crystal form.^26,27^ C30 in particular exhibits two stable rotator phases, namely RIII and RIV, which are characterized by different chain tilt directions and triclinic and monoclinic lattices, respectively.^23,28,29^

The crystallization of *n*-alkanes has also been investigated in a number of confinement geometries.^23,30,31^ The confinement in nanopores or microcapsules results in a stabilization of rotator phases in general, and transient or metastable rotator phases in particular.^30,32−35^ These studies showed that *n*-alkane phase transition temperatures are reduced inside nanopores. In *n*-alkane binary mixtures, both alkanes only cocrystallize if one alkane is no more than 22% longer than the other.^36^ Otherwise, the shorter *n*-alkane acts as a solvent for the other, with a significant reduction of the crystallization temperature of the longer alkane.^37^ While there are numerous studies investigating the organization of shorter *n*-alkanes in lipid membranes,^18,19,38−41^ we are not aware of any studies reporting on the crystallization of *n*-alkanes (or other purely hydrophobic molecules) inside lipid membranes. Filling this obvious gap will be beneficial for better understanding more complex biological problems such as the crystallization of drugs in lipid/polymer nanoparticles or the behavior of triglycerides in lipid droplets.

In this work we study the behavior of two *n*-alkanes, *n*-eicosane (C20) and *n*-triacontane (C30), inside different phospholipid membrane systems. We show that under certain conditions the crystallization of the *n*-alkanes occurs, and highlight the differences between crystallization in bulk and inside the lipid membrane for C30. To our knowledge, this work demonstrates for the first time that the crystallization of purely hydrophobic molecules inside a lipid bilayer is possible.

## Methods

### Sample Preparation

1,2-Dimyristoyl-*sn*-glycero-3-phosphocholine (DMPC, 14 carbons per acyl tail), DMPC with perdeuterated acyl tails (DMPC-d54), 1,2-dipalmitoyl-*sn*-glycero-3-phosphocholine (DPPC, 16 carbons/tail), DPPC-d62, 1-palmitoyl-2-oleoyl-*sn*-glycero-3-phosphocholine (POPC, 16/18 carbons per acyl tail) and 1,2-dioleoyl-*sn*-glycero-3-phosphocholine (DOPC, 18 carbons/tail) were obtained from Avanti Polar Lipids. Protionated and perdeuterated *n*-eicosane (C20/C20d) and *n*-triacontane (C30/C30d), methanol and chloroform were obtained from Sigma-Aldrich. Multilamellar vesicles (MLVs) of phospholipids containing *n*-eicosane or *n*-triacontane were prepared by first codissolving the lipid and 10 and 5 vol % of *n*-alkane (for C20 and C30, respectively) in chloroform. Here, the volume fraction refers to the volume of alkane per total hydrophobic volume (alkane plus acyl chains). The solvent was then evaporated under a nitrogen gas stream to produce a dry lipid film. During evaporation, the solution was subjected to sonication in a heatbath at a temperature above the bulk *n*-alkane melting and the lipid bilayer main transition temperatures. The resulting lipid films were kept at reduced pressure overnight to remove any potential residual solvent. Samples containing DMPC, POPC or DOPC were rehydrated in a high humidity atmosphere through the following procedure. Lipid films and about 2 mL of water were placed in a desiccator. The desiccator was then evacuated by briefly attaching it to a vacuum pump, until the water had degassed. Time spent in the evacuated desiccator varied between 4 h and 1 day for the different samples. This technique resulted in homogeneous hydration of about 9–20 water molecules per lipid (values determined by ^1^H MAS NMR were typically lower by 5 water molecules per lipid, compared to the weighted amounts). For the DPPC-based films, the desiccator method only resulted in about 4 water molecules per lipid. Therefore, water content in DPPC samples was adjusted by weighing the appropriate water amounts, aiming for a water content of 18 water molecules per lipid. This procedure resulted in hydration levels of *n*_w_ = 11–18, as determined by ^1^H MAS NMR. All samples were frozen before and between measurements. For NMR, samples were centrifuged into magic-angle-spinning (MAS) rotor inserts (Bruker) fitting approximately 25 μL.

### Solid-State NMR Experiments

All NMR measurements were conducted on a Bruker Avance III 400 spectrometer operating at a ^1^H Larmor frequency of 399.92 MHz (equal to a ^2^H Larmor frequency of 61.40 MHz). Data processing was conducted in Matlab. Temperature-dependent measurements were performed in between 10 and 85 °C (the exact range for each sample being determined by the main phase transition temperatures of the sample components). Sample temperature was based on setup-specific calibrations with ethylene glycol, and samples were heated and cooled at constant rates (realized by the Bruker software), usually 1 or 2 K/min. ^1^H MAS measurements were conducted at 5 kHz MAS using a standard 4 mm double-resonance MAS probe. Single-scan ^1^H spectra had a spectral width of 100 kHz. Free-induction decays (FIDs) were zero-filled, Fourier-transformed, and the lipid and water peaks were fitted with Lorentzian lineshapes to calculate the water content from the peak integrals. A 5 mm broad-band probe was used for static ^2^H NMR experiments, employing a quadrupole echo sequence.^24^ The echo delay was 40 μs, the relaxation delay was 1 s (or 50 s in a select case), and the 90° pulse width was around 4.3 or 6.0 μs. Dependent on sample composition and temperature/quadrupolar coupling strength, 1024 to 81,920 scans with a spectral width of 1 MHz were acquired for each spectrum. The FIDs were processed and Fourier-transformed using Matlab, starting from the echo maximum.

### Wide-Angle X-ray Scattering (WAXS)

WAXS measurements were performed on two samples of C20 in DPPC (10 and 25 vol % of *n*-alkane), and a DPPC reference sample. Additional water was added to the samples subjected to WAXS, resulting in a final lipid concentration of about 150 mg/mL. The dispersions were vortexed and filled into borosilicate glass capillaries (from Hilgenberg (Maisfeld, Germany); 1 mm outer diameter and 0.01 mm thickness). The experimental setup is described in detail in our previous publication and is not repeated here.^21^ 2D-Scattering profiles were measured at 22 °C and corrected for transmission and sample geometry. The scattering intensities were angular-averaged and plotted versus the scattering vector *q* before background subtraction and normalization with respect to concentration and sample volume.

### Differential Scanning Calorimetry (DSC)

DSC measurements were conducted on a PerkinElmer DSC 8000. Samples subjected to DSC measurements were limited to hydrated DMPC/C30d, hydrated DMPC, and pure C30d. Temperature sweeps were performed with heating/cooling rates of 1 and 2 °C/min, respectively. Samples were allowed to equilibrate for 5 or 10 min at the starting temperature before each run. Isothermal crystallization experiments were performed by cooling the sample from the highest temperatures to the desired crystallization temperature at 40 °C/min. Once the crystallization temperature was reached, the heat flow was monitored for a certain time (total duration ranging from 1 to 30 or 60 min). From the temperature scans, heat capacities were calculated using the pyris software (after subtracting baseline heat flows measured with empty pans), and the data was normalized to sample mass. In the mixtures, the mass of each component in the DSC sample was calculated from the amounts weighed into the mixture during preparation, assuming that no water was lost due to evaporation prior to the measurement. During the isothermal measurements, even in the absence of thermal transitions, the heat flow as a function of time only reached a constant baseline after 0.5–1 min. Therefore, such measurements were used as baselines to subtract the nonconstant heat flow recorded at short times from the data. Then, relative transition enthalpies were calculated by integrating the heat flow up to different crystallization times *t*_c_, and normalizing to the total integral. This method could only be used for bulk C30d, since in the mixtures the amount of *n*-alkane, and therefore the heat flow related to the transition events, was very small. Instead, the total duration of the crystallization step was varied, and the respective enthalpies were determined from a consecutive heating run, similar to the method used by e.g., Alamo et al.^42,43^ Using a Malvern MicroCal VP-DSC instrument, heating/cooling runs were acquired for an additional sample of 5 vol % C30d in DMPC, and a DMPC reference sample, prepared with increased amounts of water. The sample preparation was the same as described above, except for adding additional water until a lipid concentration of 10 mg/mL was reached, and vortexing and sonicating the dispersion prior to measuring. Scan rates were 1 °C/min in both heating and cooling scans ranging from 5/20–80 °C. Prescan equilibration times of 10 min were used. The reference cell was filled with pure degassed water and a water/water baseline was subtracted from each sample scan.

## Results and Discussion

This section is organized in the following way. First, we present and discuss results obtained from employing static ^2^H NMR to investigate the molecular state of C20d and C30d in phospholipid membranes under various conditions. In general, for a given C–D segment, the shape of its ^2^H NMR spectrum depends on the dynamics and geometry of the C–D bond motion.^24^ As will be shown, the different motional states of alkane molecules in fluid isotropic, fluid anisotropic, rotator, or crystal phases result in characteristic spectral shapes that enable to determine the presence and phase of *n*-alkanes inside lipid membranes. We then examine the crystallization properties of C30 in a DMPC bilayer in more detail by comparing temperature-dependent NMR and DSC measurements. Lastly, we explore the effect of sample hydration on crystallization, and compare the crystallization kinetics of pure C30 with the crystallization of C30 molecules in the membrane environment.

### C20 Becomes Trapped in the Lipid Gel Phase and Does Not Crystallize

**Figure 1:**
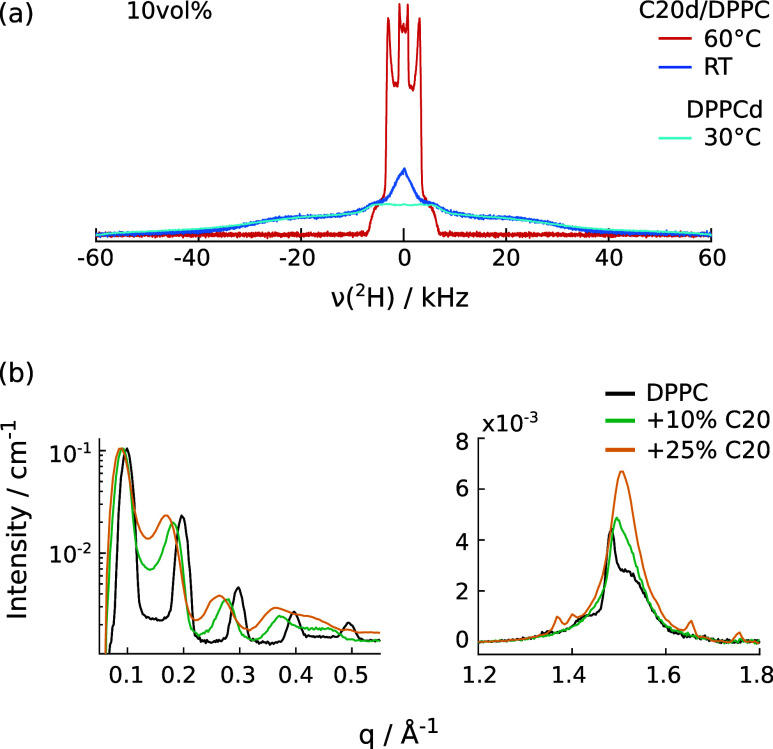
Organization of C20 in DPPC membranes at different temperatures. (a) ^2^H NMR spectra of C20d molecules in DPPC membranes (10 vol % of alkane corresponding to 0.18 C20d molecules per DPPC) at a reduced hydration of *n*_w_ = 16, and ^2^H spectrum of DPPC-d62 MLVs. The intensity of the DPPC-d62 spectrum is scaled to match the intensity of the C20d/DPPC spectra. At room temperature (25 °C), the spectral shape of C20d in DPPC resembles that of gel-phase DPPC-d62. (b) X-ray scattering profiles for fully hydrated DPPC MLV’s (at excess water) containing 0, 10, and 25 vol % of C20, measured at 22 °C.

Shorter *n*-alkanes can be incorporated into lipid membranes to larger amounts,^21^ therefore we first investigated systems with *n*-eicosane-d42 (C20d). ^2^H NMR spectra of 10 vol % C20d in a DPPC bilayer at different temperatures are shown in Figure 1a. Above the lipid and alkane melting temperatures, a spectral line shape corresponding to the anisotropic arrangement of liquid molecules is observed. This demonstrates that the C20d molecules are indeed dispersed throughout the lipid membrane as we had already shown and discussed previously.^21,44^ Upon cooling below the crystallization temperature of bulk C20d (33 °C^45^), the spectrum broadens considerably (dark blue spectrum) strongly resembling the ^2^H NMR spectra of phospholipid gel phases.^46−49^ For comparison, a spectrum acquired at 30 °C from membranes composed of perdeuterated DPPC without alkane added is shown in Figure 1 (light blue spectrum). At this temperature, the system is close to the DPPC gel-to-rippled phase transition.^46,50^ The two spectra match nearly perfectly with exception of the peak observed for C20 at the center of its spectra. We hypothesize that such feature may result from a more disordered arrangement of the alkane methyl groups in comparison to the methyl groups of perdeuterated DPPC. This notable resemblance of the ^2^H NMR spectra of C20d and perdeuterated DPPC, indicates that C20d adopts a gel-like state at room temperature, with the C20d molecules being built into the DPPC gel structure owing to their chemical similarity with the lipid acyl tails. Similar observations have previously been made for shorter *n*-alkanes in different lipid membranes.^40^

To confirm that the C20d molecules are incorporated into the gel phase, we have performed X-ray scattering experiments on DPPC multilamellar vesicles with C20 concentrations ranging from 0 to 25 vol % at a temperature below the lipid main transition. These experiments show that the presence of C20 increases the lamellar repeat distance of the bilayers in the gel phase from 63.8 ± 0.3 Å (pure DPPC) to 72.5 ± 1.8 Å (25 vol % C20), as seen by a continuous shift of the low-*q* Bragg reflections to smaller reciprocal distances (left plot in Figure 1b). In addition, C20 affects the lateral ordering of the lipid acyl tails, as evidenced by the disappearance of the shoulder of the peak at *q* = 1.5 Å^–1^ (right plot in Figure 1b). This is in line with previous observations that have been explained by an overall denser packing and the removal of acyl chain tilt.^39,51−53^ We therefore conclude that C20 is built into the DPPC gel phase, inducing a change from a tilted (observed for pure DPPC) to an untilted gel-phase L_β_. The formation of the untilted L_β_ phase is often observed in mixtures of lipids with different acyl tail lengths,^51^ or for lipids with smaller head/tail volume ratio.^54^

**Figure 2:**
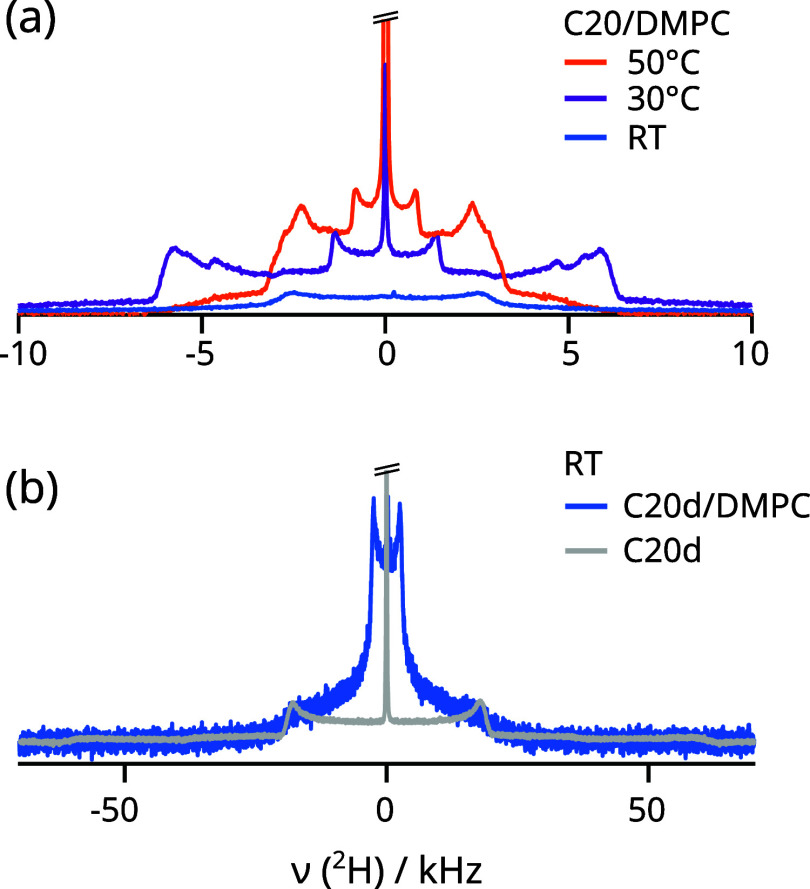
^2^H NMR spectra of 15 vol % C20d in DMPC (reduced hydration). Top: Temperature series. Spectra acquired at 50 °C (above *T*_m,DMPC_ and *T*_m,C20d_), 30 °C (above *T*_m,DMPC_ but below *T*_m,C20d_) and at room temperature (around *T*_m,DMPC_). Spectra are scaled to the same number of scans. Bottom: comparison of the room temperature spectrum (RT, approx. 23 °C) to crystalline bulk C20d at the same temperature (bulk intensity scaled to match the mixture).

In contrast to the preceding case described using DPPC, the gel-to-liquid transition of DMPC occurs at a lower temperature than the crystallization temperature for bulk C20d. Despite this difference, the behavior of C20d molecules in DMPC membranes is analogous. At 30 °C, below the crystallization temperature of the alkane and above the DMPC gel-to-liquid transition, the ^2^H spectrum of C20d does not show any sign of crystallization, and again a gel-like state is observed upon cooling the system to temperatures below the DMPC liquid-to-gel transition (about 23 °C, Figure 2b). This indicates that the crystallization temperature of C20d is reduced inside the membrane environment, similarly to what happens in mixtures composed of short and long alkanes. The same is observed in the ^2^H NMR spectra of 10 vol % C20d in DOPC (Figure S1). Since the DOPC system has a much lower temperature for the liquid-to-gel transition, we tried to reduce the temperature further to try observing crystallization. However, the DOPC/C20d sample contains a large amount of bulk *n*-alkane, making a further analysis difficult. We therefore focused on exploring the potential crystallization of C30d, having a melting temperature well above the main transition temperatures of DMPC and DPPC. In the following sections we demonstrate that C30d can indeed crystallize within a lipid membrane environment.

### C30 Crystallizes inside DMPC and POPC Membranes

**Figure 3:**
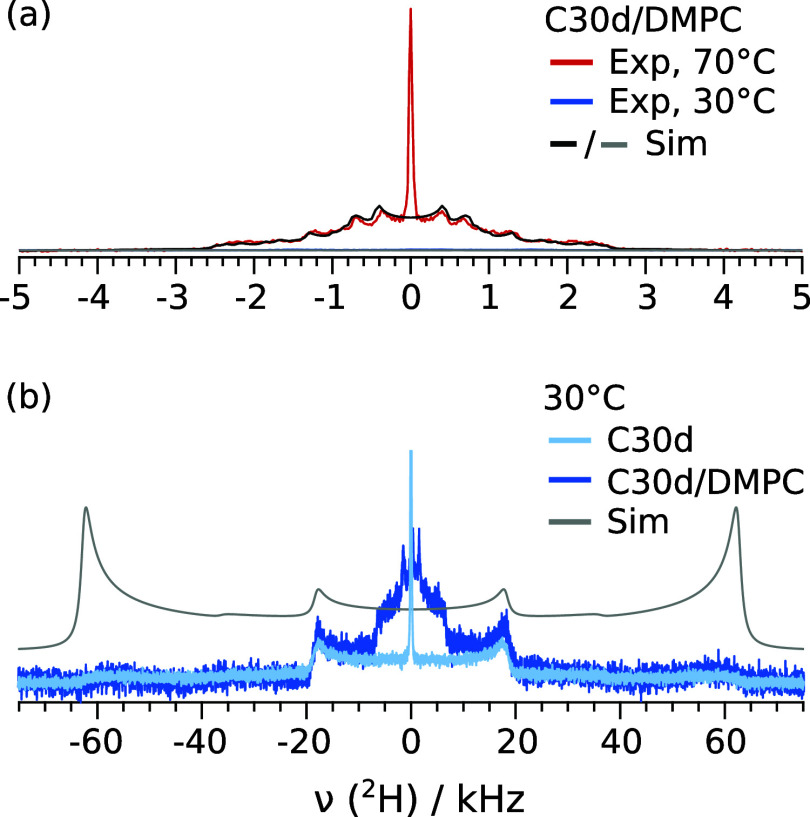
^2^H NMR spectra of 5 vol % C30d inside DMPC membranes at reduced hydration, using a quadrupole echo technique and a short recycle delay of 1 s. The frequency ranges were chosen to best visualize liquid-anisotropic and solid *n*-alkane signals, respectively. Simulated spectra are included for comparison. The simulated 70 °C spectrum was calculated by guessing the order parameter profile and summing up the resulting Pake patterns according to the procedure in our previous work.^44^ Transversal relaxation was set to *T*_2_ = 5 ms to best approximate the experimental spectrum. The crystalline spectrum was calculated based on C–D bond order parameters of 0.29 and 1.0 for methyl and methylene bonds, respectively. The overall intensity was matched to the intensity of the 70 °C spectrum, and *T*_2_ = 0.2 ms was used.

The static ^2^H NMR spectra measured from membranes containing 5 vol % of C30d in DMPC are shown in Figure 3. The high-temperature spectrum was acquired above the melting point of C30d (approximately 62 °C^45^) and originates from a superposition of Pake patterns and a narrow center peak. This narrow peak, corresponds to 10% of the spectrum (calculation described in the next section), and likely originates from a separate isotropic bulk *n*-alkane phase, i.e., C30d that did not enter the lipid membrane. Upon cooling the mixtures to 30 °C, the spectrum broadens considerably (dark blue spectrum, Figure 3). For comparison, the spectrum of crystalline bulk C30d obtained at 30 °C is also included in Figure 3b. Horns at about ±17–18 and ±60 kHz, are observed in both spectra corresponding to the expected quadrupolar splittings for the methyl and methylene groups of C30d molecules in the crystalline state.^24^ This result clearly shows that, after cooling to 30 °C, most of the C30d molecules that were initially mixed with the acyl chains at high temperature must become part of a crystalline structure. Similar quadrupolar splittings have been measured from short *n*-alkane crystals.^55^ A simulated spectra is also included in Figure 3. The outer horns of the simulated spectrum in Figure 3 are much more prominent than in the experimental spectra. Figure S2 shows that this is because the spectra in Figure 3b were acquired using a too short recycle delay of 1 s. While such a short delay is suitable for liquid *n*-alkanes,^56,57^ it is insufficient for the rigid methylene groups^48^ resulting in a loss of magnetization in comparison with the mobile methyl ends in the crystal that have a shorter spin–lattice relaxation time due to their 3-fold rotation. However, since the crystalline component can still be identified using the short recycle delay, and long recycle delays increase the measurement time beyond what is practical, we continued using a recycle delay of 1 s in this work. We have also performed experiments on samples containing C30d in POPC bilayers and the ^2^H spectra observed mirror the results obtained in DMPC (Figure S3). For both the POPC and DMPC samples containing crystallized alkane, reheating of the samples to above the *n*-alkane melting temperature leads readily to a recovery of the initial ^2^H spectrum (shown in Figure S3 for POPC/C30d). This observation suggests that C30 crystals are small and most likely located inside the lipid bilayers. Only then, the *n*-alkane molecules are able to quickly disperse throughout the membrane upon melting.

An additional component is observed in the center of the experimental spectra in Figure 3b. For bulk C30, this component appears as a residual narrow peak, indicating fast isotropic motion, likely due to impurities, e.g. shorter *n*-alkane molecules present in the sample. For C30d in DMPC, this component is much broader, and most likely originates from noncrystallized C30d molecules. A more detailed discussion of this feature and the temperature dependence of the ^2^H spectrum of C30d is provided in the following section.

In summary, our ^2^H NMR measurements show that *n*-alkanes crystallize inside lipid membranes only if the *n*-alkane melting point lies sufficiently far above the main transition temperature of the lipid membranes (e.g., C30d in DMPC, Figure 3). If the temperature difference between the two transitions is too small, upon cooling the *n*-alkane molecules become trapped in the lipid gel phase and do not crystallize (Figures 1 and 2). We observe this at very low cooling rates since the ^2^H NMR experiments performed at each temperature had a duration of 1 h or more. These results suggest that the crystallization temperature of *n*-alkanes is decreased inside a lipid membrane environment similarly to what happens when chains are dispersed in a nonstructured solvent. In order to investigate this in more detail, we performed ^1^H NMR and DSC experiments on the system C30/DMPC which are evaluated in the following section.

### Crystallization Temperature of C30 Decreases inside DMPC Bilayers

**Figure 4:**
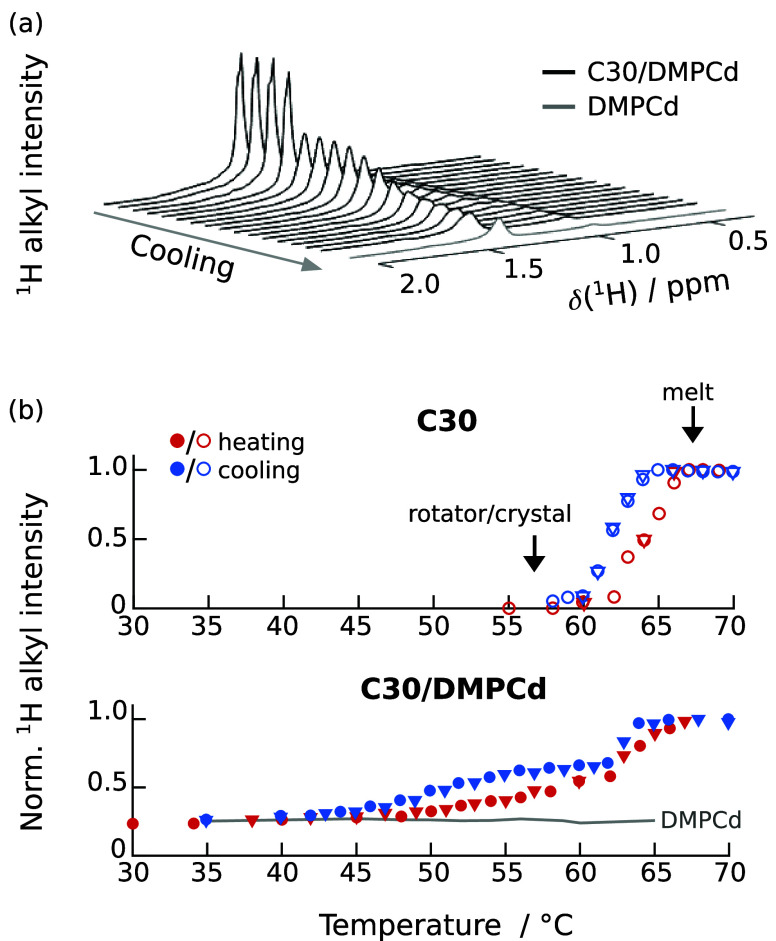
^1^H intensity in the alkyl chain spectral region of C30 as a function of temperature. (a) Alkyl spectral region of C30 in DMPC-d54 during cooling at 1 K/min. Even though the lipid is perdeuterated, some signal can be detected even when no alkane is present. (b) Normalized intensities calculated by summing all spectrum points in the ppm range displayed in (a). Upper row: bulk C30, Lower row: 5 vol % C30 in DMPCd, and pure DMPCd for reference. The different symbol shapes mark different heating/cooling runs.

In order to study the temperature dependence of the crystallization process of C30d inside DMPC membranes, we first performed ^1^H NMR measurements on mixtures of nondeuterated C30 and DMPC with per-deuterated acyl tails (DMPC-d54). The measurements were performed under magic angle spinning at a rate of 5 kHz. This allowed us to record the ^1^H NMR signal of the *n*-alkane alkyl protons during a temperature sweep. At the MAS frequency used, the ^1^H alkyl peaks can only be resolved if the alkane is in a liquid state; in the crystalline and rotator phase the peaks are broadened beyond detection due to significantly reduced chain mobility and consequent increase of the ^1^H homonuclear dipolar couplings. For the rotator phase, this observation is similar to lipid gel-phases, where reduced mobility results in increased dipole–dipole interactions and T_2_ relaxation rates.^58,59^Figure 4 shows the results of such measurements. In Figure 4a, the alkyl chain region of the ^1^H spectrum is plotted with decreasing temperature (the low-temperature spectrum of DMPC-d54/water is included also for comparison). Figure 4b, shows the normalized integrals of this spectral region, upon heating/cooling of C30 in bulk (upper plot) and in the DMPC-d54 membrane (lower plot). The melting of bulk C30 is indicated by a strong signal increase between 64 and 67 °C, which corresponds to the expected melting temperature of about 65 °C (from the rotator to the liquid phase).^28,29,60^ Following subsequent cooling, the signal intensity decreases between approximately 65 and 60 °C. According to Sirota et al., the liquid-to-rotator transition of C30 is slightly below it is melting temperature, between 64 and 65 °C, with a subsequent transition between the RIV and RIII rotator phases at 63.9–64.4 °C.^28,60^ We’d like to note here that, since the ^1^H NMR experiments were conducted under MAS, a temperature gradient of a few degrees exists within the samples. This gradient broadens the observed transitions and obstructs a more accurate comparison with literature values.

Figure 4b shows that C30 in a DMPC-d54 membrane behaves notably different from bulk C30. Upon heating, there is a gradual increase of intensity between 50 and 62 °C, after which the signal increases sharply. This two-step process becomes even more evident in the cooling runs, were we first observe a sharp decay similar to the bulk sample, followed by a second process between 55 and 45 °C.

**Figure 5:**
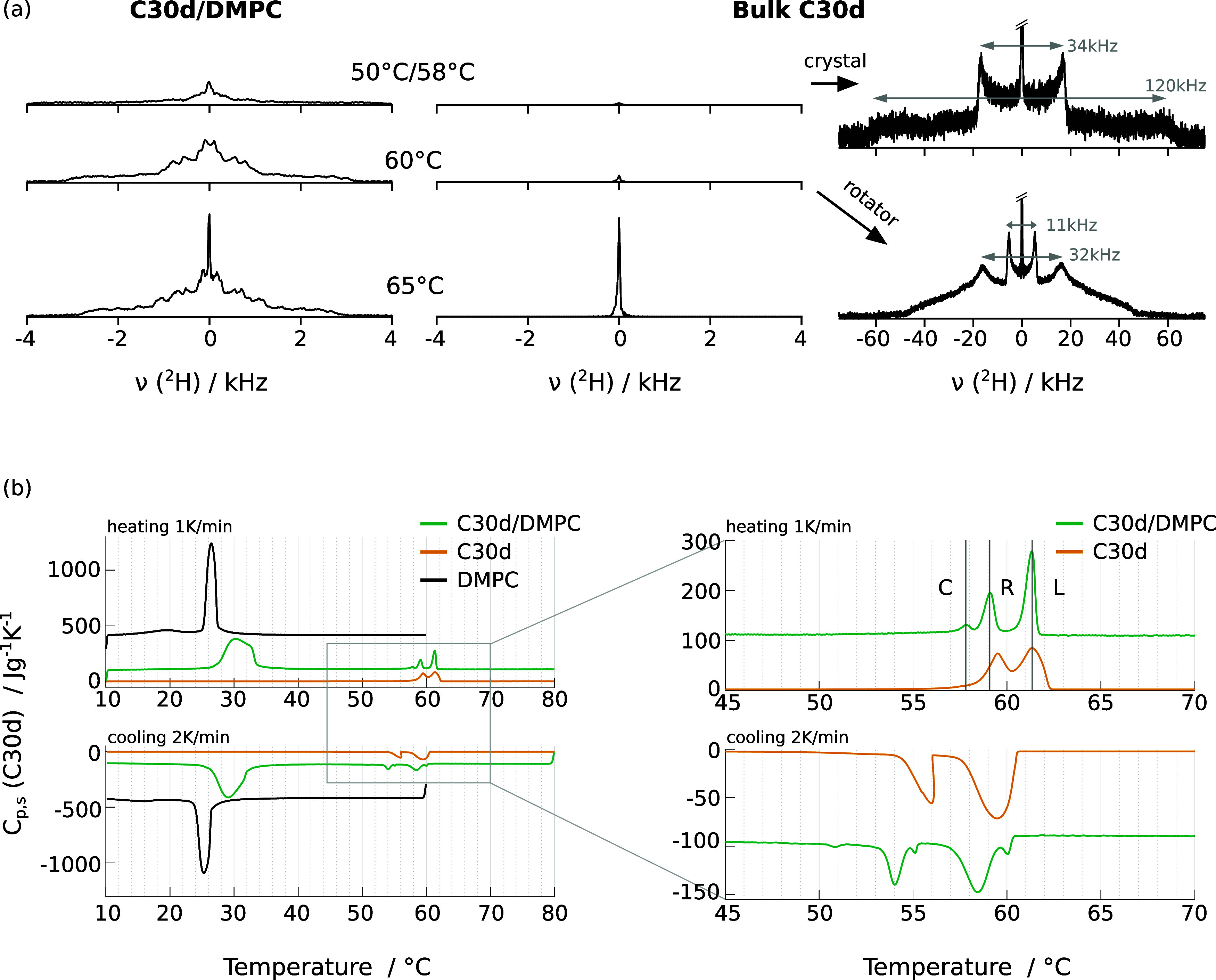
Temperature-dependent phase behavior of C30d in DMPC and in bulk. (a) ^2^H NMR spectra of 5 vol % C30d in DMPC, at reduced hydration and for three selected temperatures, compared to bulk C30d spectra (Measurement time per spectrum 35 and 17 min in the membrane and in bulk, respectively). (b) DSC heat capacities of 5 vol % C30d in DMPC, at reduced hydration, compared to DMPC (at comparable hydration) and bulk C30d. The heating rate was 1 K/min and the cooling rate was 2 K/min. Right: Zoom into the *n*-alkane phase transition region. Heat capacities are given in J K^–1^ per gram C30d and were shifted vertically to facilitate visualization. The heat capacity of DMPC/water was normalized to result in the same energy per number of DMPC molecules in the C30d/DMPC mixture.

In order to identify the alkane phases present at different stages of the cooling and heating runs, we acquired ^2^H NMR spectra of C30d in bulk and in DMPC membranes in the relevant temperature range. Selected results are shown in Figure 5a. For bulk C30d, one can clearly distinguish the crystal, rotator and liquid phases. The crystalline signal features two sets of horns, separated by approximately 34 and 120 kHz, as explained in the first part of this manuscript. The rotator phase resembles spectra of gel-phase lipids,^46−49^ while the liquid *n*-alkane results in a single peak. We obtain transition temperatures of about 60.0–60.5 °C and 62.5–64.0 °C for the crystal-to-rotator and rotator-to-liquid transition of bulk C30d upon heating, respectively (^2^H NMR spectra not shown). Upon cooling, the rotator-to-crystal transition temperature was between 60 and 58 °C. These results are in line with previous works^28,45,60,61^ and with DSC results that will be described below and shown in Figure 5b, orange curves. It is more difficult to identify exact phase transition temperatures for the C30d/phospholipid mixture. Nevertheless, the observations from ^2^H NMR measurements (Figure 5a, left column) match the ^1^H NMR results shown in Figure 4. The intensity of the ^2^H NMR spectra in the region between ±4 kHz gradually increases from about 45 to 60 °C. In this temperature range, the spectral shape observed reveals the presence of alkane molecules with anisotropic motion in the membrane environment. Additionally, at 65 °C and above, a central narrow peak becomes visible in the ^2^H NMR spectra. Since the temperature range in which this peak is observed roughly matches the temperature interval in which bulk C30d is liquid, it seems reasonable to assign this narrow signal to phase-separated bulk alkane.

In order to detect alkane crystallization, we acquired ^2^H spectra in the relevant temperature range with a much larger number of scans (up to 81,920, Figure S4). A close look at these spectra reveals the coexistence of liquid and crystalline (58 and 53 °C) or liquid and rotator phase alkane (61.0, 60.5, and 59.5 °C) in the transition range of bulk C30d.

Analysis of the ^2^H NMR spectra enabled to determine distinct fractions of C30d molecules located in different environments in the samples (Figures S5 and S6). An upper limit to the amount of bulk alkane, i.e., C30d molecules that did not enter the lipid membranes, can be estimated by a subtraction method^62,63^ illustrated in Figure S5. The spectrum of pure C30d in the melt is fitted, and then a fraction of the resulting Lorentzian line shape is subtracted from the C30d/DMPC spectrum until an intensity of nearly zero is reached in the center of the spectrum. The difference between the original and the reduced spectrum then corresponds to an upper limit for the bulk alkane fraction, and is roughly 10%. Note that this is an overestimate of the bulk alkane amount in the sample since the spectrum of anisotropic alkane should not reach zero at the center. We determined that this small amount of bulk C30d is not sufficient to account for the crystalline signal observed at e.g., 53 or 58 °C (Figure S4). This was done by comparing the ^2^H signal intensities across different temperatures, as described in more detail in the SI (Figure S6). From these calculations we obtain that, at 55 °C, more than nearly 70% of the molecules in the crystalline state must have been dispersed inside the DMPC membranes prior to crystallization. We conclude therefore that our samples contain not only two, but three different types of C30d molecules: Phase-separated bulk alkane and chains incorporated in the membrane that can be divided into those that crystallize at similar temperatures to bulk C30d, and those which remain in a liquid state at temperatures much lower than the bulk crystallization temperature.

These findings are supported by DSC heating/cooling runs shown in Figure 5b. For bulk C30d (orange curves), two peaks are observed in the heat capacity upon heating and cooling, corresponding to the crystal-rotator and rotator-liquid transitions at lower and higher temperature, respectively. Inside DMPC membranes (green curves), the crystal-to-rotator transition temperature of C30d upon heating is decreased by only 0.4 °C, while the rotator-to-liquid transition temperature is hardly affected. The transition peaks become more narrow and an additional peak is visible at 57.8 °C, prior to the original crystal-to-rotator transition. Upon cooling, the shift in the phase transition temperatures upon mixing with DMPC is more evident: the liquid-to-rotator transition is lowered by approximately 1 °C, and the rotator-to-crystal transition by 1.5–2.0 °C. Small features remain visible close to the bulk transition temperatures. Regarding the properties of the DMPC membranes, the main transition is broadened and shifted to higher temperatures upon addition of C30d, and the pretransition can no longer be observed in the mixture (green vs black curves). Notably, the DSC results show no indication of a broad transition in the 45–55 °C interval, proving that no crystallization occurs in this temperature range.

Combined, our ^2^H NMR and DSC results can be interpreted in the following way. As seen from the ^2^H NMR spectra (Figures 3a, 5a and S5), at high temperatures (*T* ≤ 62 °C), the C30d/DMPC sample contains *n*-alkane mixed with the lipid and a small amount of bulk alkane (less than 10% of alkane molecules). If the sample is cooled, bulk C30d crystallizes first, followed soon after by a fraction of the C30d molecules inside the DMPC membrane as shown by the DSC curves (Figure 5b). The fraction of crystallizing chains can be determined by comparing the DSC transition enthalpies of the mixture with the bulk transition enthalpies. By integrating the (rotator-to-liquid) melting peak we find that the melting enthalpy of C30d in DMPC amounts to roughly 81 ± 3% of the bulk C30d melting enthalpy. Therefore, 19 ± 3% of C30d molecules do not crystallize in the mixture. These noncrystallizing chains will be discussed further below.

Before the actual crystallization, both bulk C30d and the C30d molecules incorporated into the lipid membranes transition through a rotator phase (Figure 5b). According to literature, the high-temperature peak in the heat capacity upon cooling should signify the transition from liquid to the RIV rotator phase.^28,29,60^ The RIV phase is then expected to transform into the RIII phase if the sample is cooled further. However, we do not see any indication of a rotator–rotator transition for bulk C30d. This is not unexpected, since the latent heat involved in this transition would be very small.^29,60,64^ Consequently, we cannot determine which rotator phase is adopted at any given temperature, and whether both rotator phases can be formed in the lipid membrane.

Upon further cooling, at about 54 °C (according to DSC, green curve in Figure 5b), the C30d molecules that had initially anisotropic motions in the lipid membrane, and that formed a rotator phase, crystallize. Notably, this transition happens at a different temperature than for bulk C30d. With ^2^H NMR, the crystallization is already observed at 58 °C (Figure S4). This discrepancy between DSC and NMR is easily explained by the acquisition time needed for the ^2^H NMR spectra presented in Figure S4, ranging from 14 to 24 h, resulting in a very small, effective cooling rate. DSC scans however were conducted at cooling rates of 2 °C/min and thus the DSC transition temperatures can be expected to be lower than those obtained from ^2^H NMR.^65,66^ Furthermore, our ^2^H NMR results have a relatively low temperature resolution (about ±1 °C). This makes it difficult to detect slight shifts of the crystallization temperature such as the one between bulk and incorporated C30.

If C30d inside DMPC membranes crystallizes at only slightly lower temperatures than bulk C30d, the anisotropic liquid C30d component which is observed between approximately 45 and 60 °C in the ^2^H spectra (Figure 5a) must originate from *n*-alkane molecules inside the membrane which do not crystallize. We suspect that these noncrystallizing *n*-alkane molecules are built into the DMPC phase, similar to C20 in DMPC. Such a coassociation of alkane and lipid is supported by the increased main transition temperature of DMPC in the presence of C30d seen with DSC (Figure 5b). As mentioned previously, a shift of the lipid main transition temperature is expected when adding longer *n*-alkanes to saturated phospholipid bilayers. The loss of the pretransition temperature has also been observed previously in mixtures of C12 or C14 with DPPC.^39,53^ It should however be noted that, while the DMPC and C30d/DMPC samples compared in Figure 5b were prepared in an identical manner, hydration somewhat differs between the samples. After preparation, the samples contained 22.5 and 12.5 water molecules per lipid for DMPC and C30d/DMPC, respectively. Since lipid acyl chain ordering increases with decreasing water content below approximately 25–30 water molecules per lipid,^67^ the shift of the DMPC main transition partially relates to a decrease in hydration. However, at the estimated water concentration, we would not expect such a large shift of the main transition temperature, and the pretransition should still exist (in the absence of alkane).^68,69^ Therefore, we suggest that the shift of the lipid main transition demonstrates an association between alkane and lipid molecules.

Our interpretation so far does not fully explain the continuous NMR signal decrease between 60 and 45 °C (Figures 4 and 5a). This signal decrease must be related to the noncrystallizing C30d in the membrane, since all other *n*-alkane molecules should already be crystalline at this point. However, the associated lipid/alkane gel transition does not happen until about 35 °C. Generally, lipid ordering decreases with increasing temperature even in the liquid-crystalline phase,^51^ and lipid dynamics are also affected irrespective of phase transitions.^70^ It is possible that changing lipid dynamics affect the *n*-alkane NMR relaxation rates sufficiently to result in intermediate-motion lineshapes and signal loss even before the actual phase transition.

### Increase of Membrane Hydration Leads to Homogeneous Nucleation

**Figure 6:**
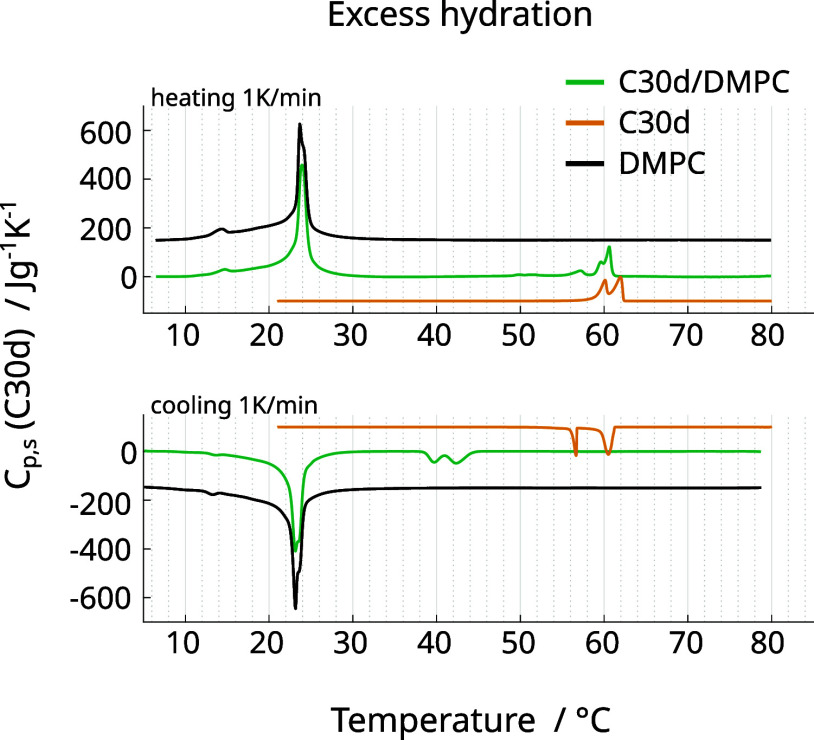
DSC heat capacities in excess water conditions. Heating and cooling runs (1 K/min) of 5 vol % C30d in DMPC (lipid concentration 10 mg/mL) are compared to DMPC (at comparable hydration) and bulk C30d. Heat capacities are given in J K^–1^ per gram C30d and were shifted along *y* to not overlap. The heat capacity of DMPC/water was normalized to result in the same energy per number of DMPC molecules in the C30d/DMPC mixture.

Finally, we have investigated whether the crystallization of C30d inside DMPC is affected by the low amount of water in our systems. Figure 6 shows DSC heating and cooling runs of 5 vol % C30d in DMPC in excess water conditions. There are two important differences compared to the reduced hydration sample: First, the DMPC main transition is not at all affected by the presence of C30d (black vs green curves). Second, the cooling transitions of C30d are shifted to much lower temperatures (orange vs green curves): the liquid-to-rotator and rotator-to-crystal transition occur at 19.5 and 20.5 °C below the bulk C30d melting temperatures, respectively. Such a strong hysteresis between heating and cooling transitions is evidence for homogeneous nucleation of C30d.^27,71−74^ We previously showed that under excess water conditions, *n*-alkanes induce the formation of smaller lipid structures (vesicles or maybe micelles with alkane droplets).^21^ Confinement in such small structures could explain why homogeneous nucleation is possible in this system. Furthermore, the unaffected lipid transition supports this interpretation, since the alkane would be spatially separated from most of the lipid in this scenario.

It is interesting that we still observe two peaks in the DSC cooling runs, showing that a rotator phase is adopted prior to crystallization of C30d. Previously, the role of the rotator phase upon homogeneous nucleation was not clear.^27^ Rotator phases were observed in droplets of odd *n*-alkanes between 15 and 19 carbons, and both the liquid-to-rotator and rotator-to-crystal transition temperatures were decreased notably. However, the liquid-to-rotator transition temperature was affected more strongly, reducing the interval in which the rotator phase could be observed.^72,75^ For odd and even *n*-alkanes between C20 and C32, rotator phases were observed during heating, but not upon cooling.^71,72,74,76^ Consequently, it was argued that these *n*-alkanes crystallize directly, without transitioning through a rotator phase, upon homogeneous crystallization. It has also been found that the interface between *n*-alkane and the confining material (e.g., surfactant or polymer microcapsules) can induce surface heterogeneous nucleation.^77−81^ In such systems the freezing temperatures are not reduced as much as in the case of homogeneous nucleation, and rotator phases occur if the surfactant hydrocarbon tails are of similar length as the alkane.^77,78^ Our measurements show a rotator phase even during cooling, as well as significant undercoolings for both the liquid-to-rotator and rotator-to-crystal transition (Figure 6). It seems reasonable that the lipid acyl tails can induce surface heterogeneous nucleation similar to the surfactants mentioned above. Weidinger et al. argue that such nucleation does not necessarily have to result in smaller undercoolings, since the number of molecules exposed to the surface, and therefore available for nucleation, is comparatively small.^75^ Possibly, the liquid-to-rotator transition of C30d in our samples is indeed heterogeneous, and therefore still visible in the DSC scans. Only the rotator-to-crystal transition might be truly homogeneous, similar to mixtures of C18/C19 investigated by Jiang et al.^79^ or observations by Kovacik et al. on C16 in surfactant emulsion droplets.^82^

### Crystallization Kinetics of C30d Are Slower in the Membrane Environment

**Figure 7:**
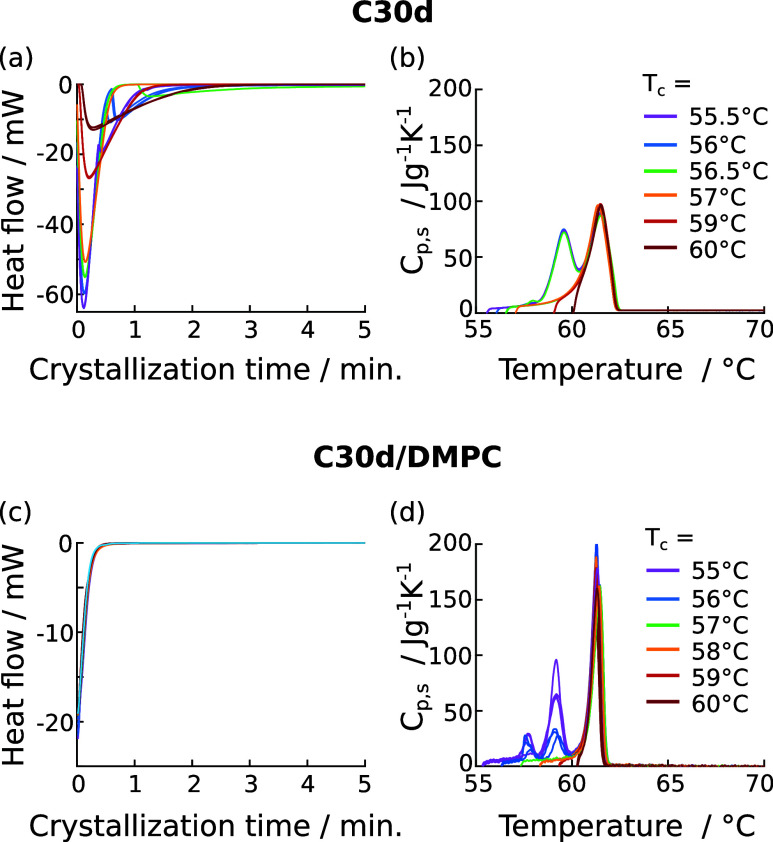
Isothermal crystallization of bulk C30d and of C30d mixed with DMPC membranes. (a) Corrected heat flow during isothermal crystallization of C30d at different crystallization temperatures *T*_c_. (b) Heat capacity of C30d during heating directly after the isothermal crystallization step shown in (a). Heating rate 1 K/min. (c, d) The same as in (a, b) but for 5 vol % C30d in DMPC. Due to the low amount of alkane in the mixture, the artifact at very short crystallization times could not be subtracted reliably, causing the heat flow during isothermal crystallization to look the same for all measurements.

In order to compare the crystallization kinetics of C30d in the membrane and in bulk, we performed DSC isothermal crystallization experiments. The heat flow recorded during isothermal crystallization for C30d and C30d/DMPC samples is plotted in Figure 7a,c. For bulk C30d, one or two transition events can be identified per temperature. For C30d in DMPC however, the heat flow curves look nearly identical for all temperatures. The initial increase in heat flow observed for this sample is not a transition event but simply the stabilization of the heat flow after cooling. This artifact could not be removed reliably, since the amount of *n*-alkane in the sample was very small. However, alkane crystallization clearly takes place in the C30d/DMPC mixture since melting peaks are observed when the sample is heated again, directly after the crystallization step (Figure 7d). For isothermal processes above 56.5 °C, both bulk and mixed C30d show a similar behavior upon remelting since only a single peak at 61.5/61.3 °C is observed. The presence of only one peak in the heat capacity profile upon heating shows that C30d remains in the rotator phase at the respective temperatures and does not crystallize. At lower temperatures, crystallization does take place after some time, as expected. In this case, the heat capacity profiles show distinct behavior for bulk C30d and C30d in DMPC membranes. For bulk C30d only two peaks are observed upon heating, while for C30d molecules in DMPC membranes three distinct melting peaks are observed.

**Figure 8:**
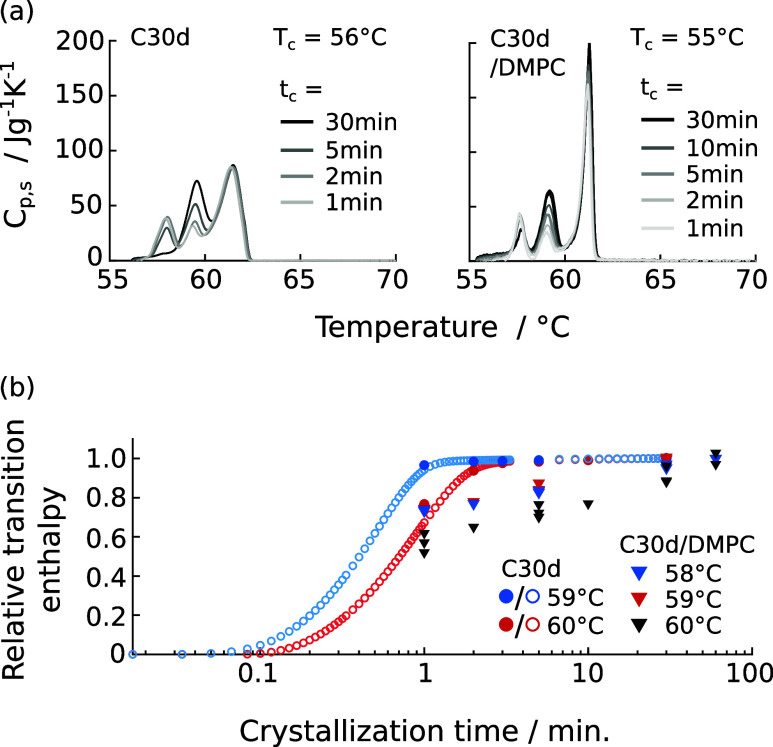
Effect of varying the duration *t*_c_ of the isothermal process for both bulk C30d and C30d mixed with DMPC membranes. (a) Heat capacity of C30d during heating runs conducted directly after isothermal crystallization at temperature *T*_c_. (b) Relative transition enthalpy of the liquid-to-rotator transition as a function of crystallization time. For bulk C30d, values were obtained directly during isothermal crystallization (open symbols) and by varying the crystallization time and comparing the subsequent melting enthalpies (filled symbols). For bulk C30d, the relative enthalpy is calculated with respect to the enthalpy after 30 min of crystallization. For C30d in DMPC, the relative enthalpy is calculated with respect to the integrated enthalpy after 60 min of crystallization. For the mixture, the liquid-to-rotator transition temperature of C30d is 1 °C below the transition in bulk, which is considered in the color-coding.

Since the heat flow for C30d in DMPC could not be measured during the isothermal crystallization itself, we varied the duration of the isothermal step between 1 and 60 min. Then, we heated the samples again and compared the heat capacities and transition enthalpies upon melting for various crystallization durations. Figure 8a shows the effect of variable crystallization time on the heat capacities during melting for bulk and mixed C30d. Similar to the heating/cooling runs shown in Figure 5b, mixing of C30d with DMPC results in a third peak upon melting (Figure 8a, right). Interestingly, if shorter crystallization times are used, this additional peak is also observed in bulk C30d (at 57.9–58.0 °C, Figure 8a, left). For both samples, the intensity of this peak decreases with crystallization time. Simultaneously, the peak at approximately 59 °C increases in intensity. This observation confirms that the additional peak corresponds to the melting of an intermediate crystal phase which is formed during the transition from the rotator to the low-temperature crystal phase. To our knowledge, C30 is not known to have multiple crystal phases,^22,29^ and we did not see any indication of an additional transition in the standard DSC cooling runs (Figure 5b, orange curve). However, Alamo et al. previously explained a similar observation in C_168_H_338_ by a crystal-thickening process.^42,43^ Indeed, it was proposed that *n*-alkanes as short as C25 may first crystallize with nonaligned chain ends,^27,83,84^ effectively reducing the crystal thickness until the crystal-perfecting process is complete. Such thin crystals would be expected to melt at lower temperatures, possibly explaining the additional peak. Irrespective of its nature, this intermediate crystal form seems to be stabilized in DMPC membranes.

Variation of the duration of the isotherm process was also used to determine the speed of the liquid-to-rotator transition. Figure 8(b) shows the relative transition enthalpies as a function of total crystallization time, obtained from integrating the heat capacity on reheating after isothermal crystallization. These relative transition enthalpies represent the fraction of *n*-alkane molecules in the rotator phase at a given time. For bulk C30d, the transition enthalpies obtained from integrating the heatflow during the long isothermal crystallization steps are plotted as a reference (open circles), and show that both methods are in good agreement. In bulk both the liquid-to-rotator and rotator-to-crystal transition occur faster at lower crystallization temperatures (red vs blue circles, see also Figure S7). Consequently, *n*-alkane diffusion coefficients should be of low relevance in this system. For C30d in DMPC, our data is not precise enough to make such a claim. However, the formation of the rotator phase appears to be a two-step process: Initial “gelation” of preorganized *n*-alkane chains occurs as fast as in bulk, as evidenced by the high relative transition enthalpy after only 1 min of crystallization (triangles in Figure 8b). The following slow increase in melting enthalpy suggest that additional molecules are added to the rotator-phase nuclei with time. Notably, the transition enthalpy measured in the mixture would also include some bulk alkane, as described above. By subtracting a fixed fraction of the bulk transition enthalpy at each time point, the relative transition enthalpy of only the mixed alkane can be estimated. Evidently (Figure S8), assuming 20% of bulk C30d in the sample, the relative transition enthalpy still reaches a value of 0.66 after 1 min, confirming that initial formation of the rotator phase is indeed very fast.

## Conclusions

We have investigated the crystallizability of *n*-eicosane and *n*-triacontane in phospholipid bilayers. Our results suggest that there is a relation between the difference in bulk melting temperatures (alkane and lipid) and the crystallizability of the *n*-alkane. In DMPC and DPPC, C20 coassociated with the lipid acyl tails and therefore transitioned to a gel-like phase together with the lipid. C20 did appear to crystallize in DOPC bilayers, however due to the large fraction of bulk alkane in this sample it was impossible to validate our findings in the same manner as for the other investigated mixtures. C30 was able to crystallize inside DMPC and POPC bilayers. In a sample containing 5 vol % of C30 in DMPC, and only low amounts of water, about 85–90% of the alkane molecules were incorporated into the bilayer. The remaining chains did not mix with the lipid at all. Of the incorporated *n*-alkane, approximately 75–80% crystallized at temperatures slightly below the bulk crystallization temperature. The noncrystallizing chains were instead built into the lipid phase, similar to C20. Inside the bilayer, C30 also crystallized via at least one rotator phase, and an intermediate crystal phase was stabilized compared to the bulk sample. With regards to crystallization kinetics, the formation of the rotator phase was observed to consist of two steps in the membrane: First, preorganized *n*-alkane chains transitioned as fast as in bulk, but further addition of molecules to the rotator-phase crystal was slowed significantly. This finding suggests that the molecular arrangement of the *n*-alkane in the lipid bilayer strongly influences the crystallization process.

In excess water conditions, we instead observed a strong reduction of transition temperatures upon cooling, suggesting that C30d is confined to smaller droplets and crystallizes homogeneously.

This work marks the first observation of crystallization of purely hydrophobic molecules inside the hydrophobic core of lipid membranes. While the investigated *n*-alkanes are still far shorter than actual polymers, this study provides a first impression of what to expect from the crystallization of long hydrophobic chains inside lipid bilayers. Furthermore, our results motivate studying more biologically relevant systems such as crystallizable drugs or triglycerides inside model cell membranes.

## Supporting Information Available

Additional ^2^H NMR spectra as a function of temperature; one analysis scheme to estimate the amount of bulk alkane in samples; and relative transition enthalpies as a function of crystallization time (PDF)
