# Supplementary material for: Crystallization of n-Alkanes under Anisotropic Nanoconfinement in Lipid Bilayers
Source: J Phys Chem B. 2024 Dec 19;129(1):435–46. doi: 10.1021/acs.jpcb.4c04332 (PMC11726633; doi:10.1021/acs.jpcb.4c04332)
Supplement: Supplementary file 1 — jp4c04332_si_001.pdf [file jp4c04332_si_001.pdf]

Crystallization of *n*-Alkanes under Anisotropic Nano-Confinement in Lipid Bilayers

Anika Wurl,[1] Maria Ott,[2] Christian Schwieger,[3] and Tiago M. Ferreira[1,4,*]

[1] *NMR group - Institute for Physics, Martin Luther University Halle-Wittenberg, Germany*
[2] *Department of Biotechnology and Biochemistry, Martin Luther University Halle-Wittenberg, Halle (Saale), Germany*
[3] *Institute of Chemistry, Martin Luther University Halle-Wittenberg, Halle (Saale), Germany*
[4] *CiQUS and Department of Physical Chemistry, University of Santiago de Compostela, Spain*

(Dated: November 22, 2024)

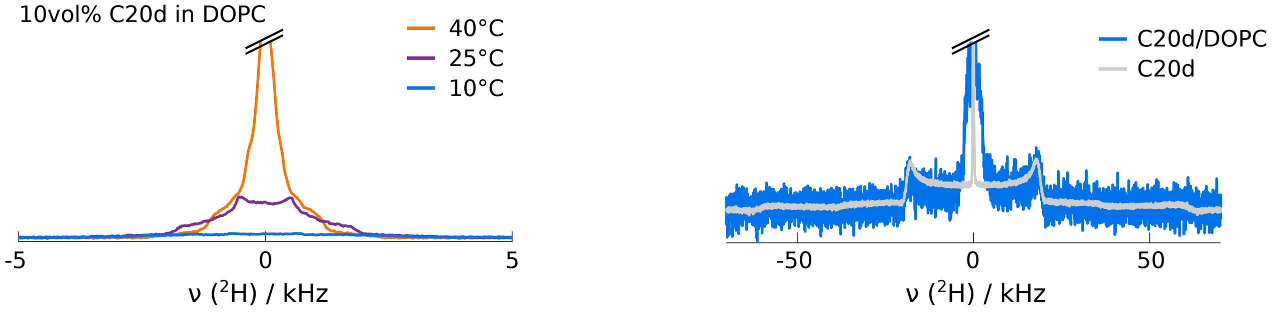

FIG. S1: ^{2}H NMR spectra of 10 vol% C20d in DOPC (reduced hydration). Left: spectra acquired above (40°C), below (25°C) and far below (10°C) the crystallization temperature of bulk C20d. Spectra scaled to the same number of scans. Right: comparison of the 10°C spectrum to crystalline bulk C20d at 23°C (bulk intensity scaled to match the mixture).

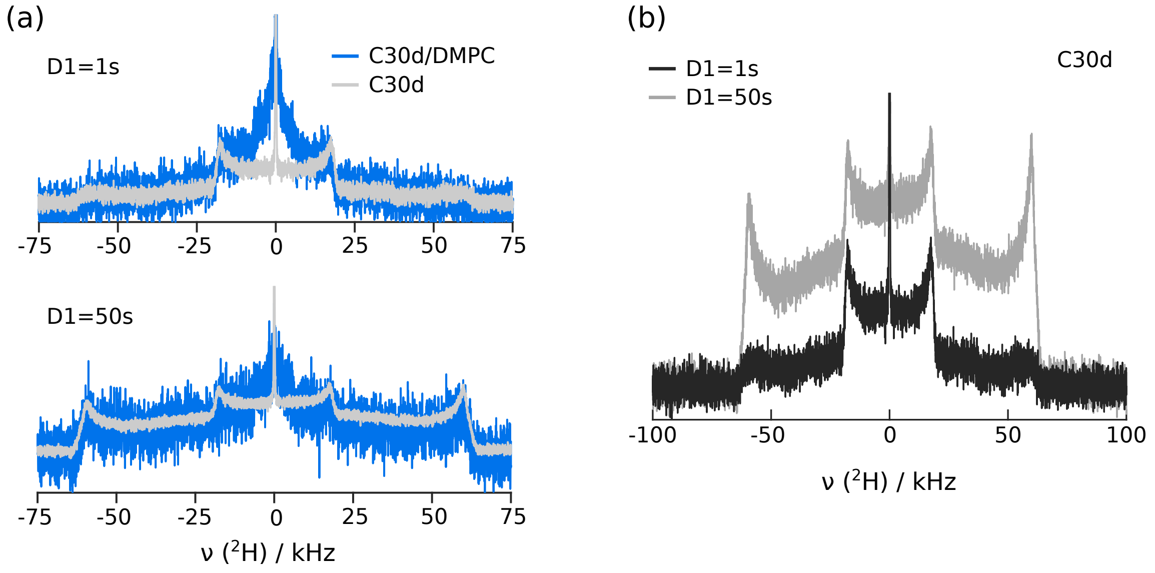

FIG. S2: Effect or recycle delay on the ^{2}H spectral shape. (a) Spectra of 5 vol% C30d in DMPC and bulk C30d, comparing long and short recycle delays (D1=1s and 50s). (b) Spectra of bulk C30d acquired using different recycle delays D1 (but otherwise identical experimental settings).

* Electronic address: tiago.mendes.ferreira@usc.es

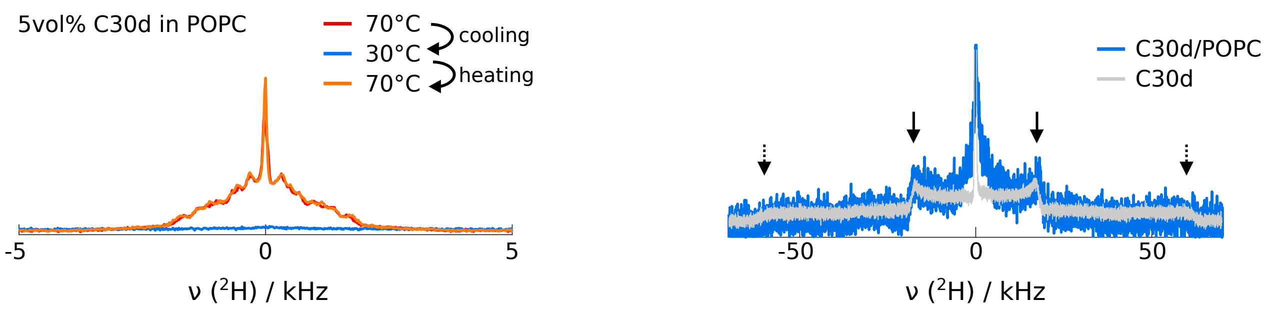

FIG. S3: ^{2}H NMR spectra of 5 vol% C30d in POPC (reduced hydration). Left: Temperature series; 70°C (above the melting temperature of C30d), then cooled to 30°C (far below the crystallization temperature of C30d), and heated again to 70°C. Spectra scaled to the same number of scans. Right: comparison of the 30°C spectrum to crystalline bulk C30d at the same temperature.

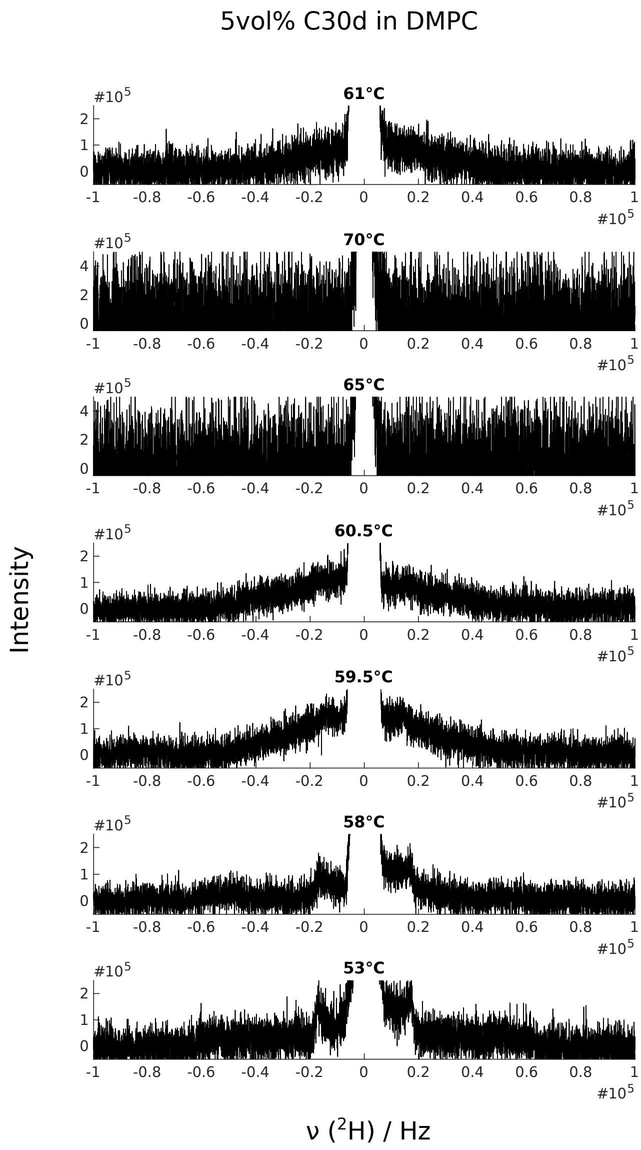

FIG. S4: Broad signal component of the ^{2}H NMR spectra of 5 vol% C30d in DMPC, at different temperatures. Spectra were acquired sequentially, from top to bottom, and are scaled to the same number of scans (NS=2048), such that the intensities are comparable.

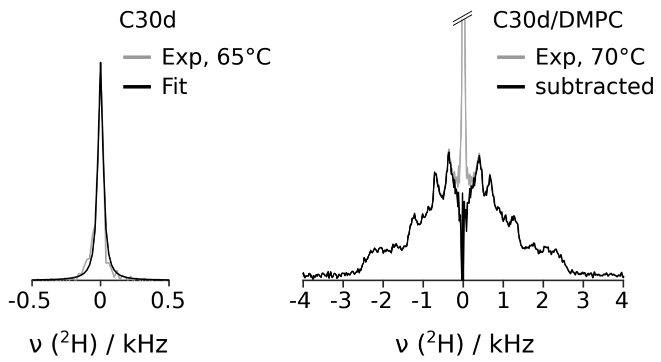

FIG. S5: Subtraction method for determining the bulk alkane fraction in C30d/DMPC samples. Left: Fit of the bulk C30d signal with a single Lorentzian. Right: Subtraction of the scaled, fitted bulk signal from the C30d/DMPC spectrum.

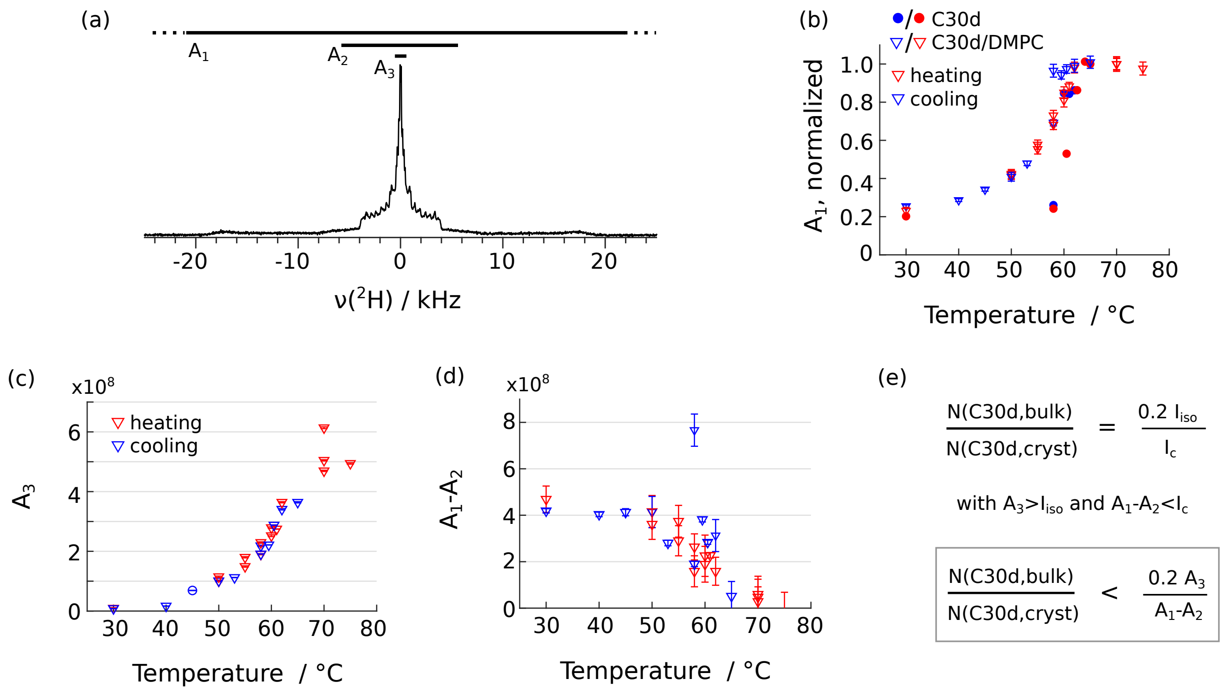

FIG. S6: Areas of ^{2}H NMR spectra, A_i, calculated by summation of spectrum points in the intervals [-100kHz,100kHz] (A_1), [-6kHz,6kHz] (A_2) and [-100Hz,100Hz] (A_3), as functions of temperature. Only spectra acquired with a relaxation delay of 1 s were considered. (a) Exemplary spectrum acquired at 50°C. (b) Normalized areas of the full spectra, including crystalline, rotator and liquid C30d chains. When only crystalline chains are present (30°C), the area is only 20 % of area at 65-75°C due to significant relaxation effects for the higher couplings. (c),(d) Areas used to estimate the amount of bulk C30d molecules relative to all crystallizing molecules. An upper limit of 31±6 % was calculated, using the formulas and approximations shown in (e). The factor 0.2 is due to the effects of relaxation on the measured areas, see (b). A_3 will always be larger than the bulk signal intensity I_{iso}, since there will be a non-zero intensity in the center of the spectrum, even if no bulk alkane is present. Similarly, A_1-A_2 will always be smaller than the total crystalline intensity I_c, since part of the crystalline component is also cut off when subtracting A_2. We used A_3(T=70°C), when all bulk alkane is liquid, and A_1-A_2(T=55°), when all crystallizing molecules should be crystalline. While this is a fairly rough estimate, it clearly shows that bulk alkane alone is not sufficient to account for the broad spectral components shown in figure S4.

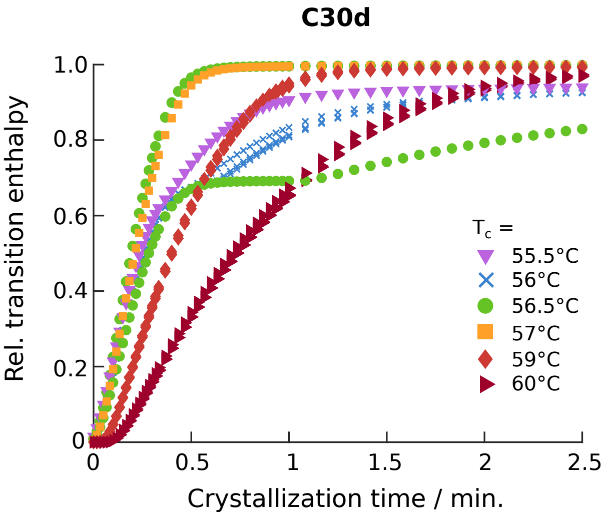

FIG. S7: Relative transition enthalpy of C30d during isothermal crystallization, obtained by integrating the heat flow data and normalizing to the total integral (up to 30 min).

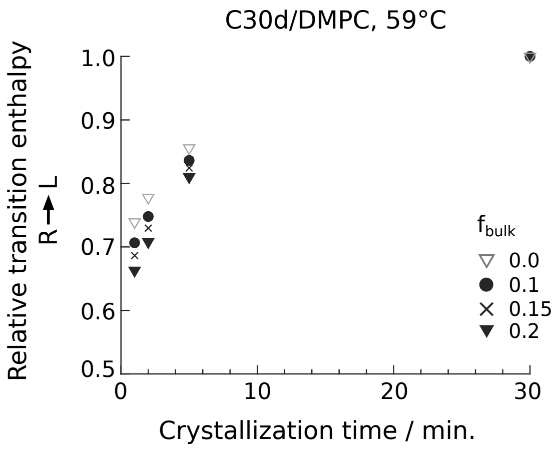

FIG. S8: Rotator-to-liquid transition enthalpy of C30d in DMPC, corrected for bulk alkane. Assuming a constant fraction of bulk C30d, f_{bulk}, the measured transition enthalpy was reduced based on the bulk transition enthalpy at the same crystallization temperature/time.
